# Supplementary material for: Role and mechanism of NCAPD3 in promoting malignant behaviors in gastric cancer
Source: Front Pharmacol. 2024 Apr 22;15:1341039. doi: 10.3389/fphar.2024.1341039 (PMC11070777; doi:10.3389/fphar.2024.1341039)
Supplement: Supplementary file 11 [file DataSheet2.ZIP › GSEA/Transcipt factor/my_analysis.Gsea.1599461814734/FREAC7_01.html]

Details for gene set FREAC7\_01[GSEA]

|  || Dataset | filtered\_dataset.sample\_info.cls#WT\_versus\_NCAPD3\_MUT |
| Phenotype | sample\_info.cls#WT\_versus\_NCAPD3\_MUT |
| Upregulated in class | NCAPD3\_MUT |
| GeneSet | FREAC7\_01 |
| Enrichment Score (ES) | -0.4978901 |
| Normalized Enrichment Score (NES) | -2.0533607 |
| Nominal p-value | 0.0023094688 |
| FDR q-value | 0.053716555 |
| FWER p-Value | 0.448 |
Table: GSEA Results Summary

  

Fig 1: Enrichment plot: FREAC7\_01      
 Profile of the Running ES Score & Positions of GeneSet Members on the Rank Ordered List

  

| SYMBOL | TITLE | RANK IN GENE LIST | RANK METRIC SCORE | RUNNING ES | CORE ENRICHMENT || 1 | 4281 | MID1 | 294 | 0.595 | -0.1407 | No |
| 2 | 2920 | CXCL2 | 454 | 0.501 | -0.1957 | No |
| 3 | 79047 | KCTD15 | 469 | 0.493 | -0.1478 | No |
| 4 | 23767 | FLRT3 | 781 | 0.333 | -0.3314 | No |
| 5 | 54206 | ERRFI1 | 849 | -0.229 | -0.3525 | No |
| 6 | 80315 | CPEB4 | 1053 | -0.448 | -0.4453 | Yes |
| 7 | 8727 | CTNNAL1 | 1106 | -0.482 | -0.4259 | Yes |
| 8 | 92014 | SLC25A51 | 1155 | -0.519 | -0.3993 | Yes |
| 9 | 2119 | ETV5 | 1176 | -0.545 | -0.3496 | Yes |
| 10 | 153222 | CREBRF | 1187 | -0.559 | -0.2911 | Yes |
| 11 | 26137 | ZBTB20 | 1244 | -0.622 | -0.2581 | Yes |
| 12 | 55691 | FRMD4A | 1295 | -0.689 | -0.2129 | Yes |
| 13 | 3084 | NRG1 | 1320 | -0.739 | -0.1433 | Yes |
| 14 | 4734 | NEDD4 | 1369 | -0.856 | -0.0771 | Yes |
| 15 | 9966 | TNFSF15 | 1376 | -0.900 | 0.0244 | Yes |
Table: GSEA details [plain text format]

  

Fig 2: FREAC7\_01      
 Blue-Pink O' Gram in the Space of the Analyzed GeneSet

  

Fig 3: FREAC7\_01: Random ES distribution      
 Gene set null distribution of ES for **FREAC7\_01**
